# SOX17 expression in tumor‐penetrating vessels in relation to CD8 ^+^ T‐cell infiltration in cancer stroma niches

**DOI:** 10.1111/1759-7714.15464

**Published:** 2024-10-09

**Authors:** Hirotaka Yamamoto, Yuki Hanamatsu, Chiemi Saigo, Tamotsu Takeuchi, Hisashi Iwata

**Affiliations:** ^1^ Department of General Thoracic Surgery Gifu University Hospital Gifu Japan; ^2^ Department of Pathology and Translational Research Gifu Medical School of Medicine Gifu Japan; ^3^ Center for One Medicine Innovative Translational Research COMIT, Gifu University Gifu Japan; ^4^ The United Graduate School of Drug Discovery and Medical Information Sciences Gifu University Gifu Japan

**Keywords:** CD8^+^ T cells, lung adenocarcinoma, prognosis, SOX17, tumor endothelial cells

## Abstract

**Introduction:**

Sex‐determining region Y‐related high‐mobility group box 17 protein (SOX17), a proangiogenic transcription factor, is specifically expressed in tumor endothelial cells (TECs) of implanted Lewis lung carcinoma. However, the expression profile of SOX17 is largely unknown in human lung cancer. We aimed to elucidate SOX17 expression in cancer cells and the tumor microenvironment of lung adenocarcinoma.

**Methods:**

In the present study, we examined SOX17 expression in whole‐tissue specimens of 83 lung adenocarcinomas by immunohistochemistry.

**Results:**

SOX17 immunoreactivity was minimal in lung adenocarcinoma cells, except in five non‐mucinous adenocarcinomas in situ. SOX17 was also expressed in cultured A549 lung adenocarcinoma cells, which is widely used as a model of malignant alveolar type II epithelial cells. Notably, SOX17 immunoreactivity was found in endothelial cells of tumor‐penetrating vessels in 19 of 83 lung adenocarcinoma tissue specimens, with statistical significance to stromal infiltration of CD8^+^ T cells (*p <* 0.01) but was not associated with the number of tertiary lymph nodes. Although not statistically significant, SOX17 immunoreactivity was related to favorable patient outcomes.

**Conclusion:**

Our findings indicate that SOX17 might play a pleiotropic role in lung adenocarcinoma in cancer cells and stromal niches. SOX17‐mediated CD8^+^ T‐cell‐rich tumor microenvironment might attract interest in improving the effect of cancer immunotherapy.

## Graphical abstract

Sex‐determining region Y‐related high‐mobility group box 17 protein (SOX17) immunoreactivity was observed in TECs with high endothelial venule (HEV) features, including a plump, almost cuboidal appearance of varying degrees (a). SOX17 immunoreactivity was nonuniform and rather heterogenous even in adjacent endothelial cells (black and white arrows indicate robust and weak SOX17 immunoreactivity, respectively). The numerous red‐stained CD8^+^ T lymphocytes present around the SOX17‐positive HEVs are notable.

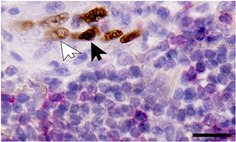

## INTRODUCTION

Lung adenocarcinoma (LUAD) is among the most common subtypes of lung cancer.^1^ The prognosis remains unfavorable for many patients, especially those with advanced LUAD.^2^ Although immunotherapy is promising for patients with advanced LUAD,^3^ less than half of these patients treated with immunotherapy show a response, and a smaller proportion achieve a long survival.^4^ Insufficient intratumoral CD8^+^ T lymphocytes result in poor response to cancer immunotherapy.^5^ Therefore, the underlying mechanism to generate a CD8^+^ T‐cell‐rich tumor microenvironment (TME) has attracted interest in improving the effect of cancer immunotherapy.

Tumor vasculature is important for the tumor immune system and facilitates the influx of immune cells into the TME,^6^ where neovascularization is principally orchestrated by tumor endothelial cells (TECs).^7^ Sex‐determining region Y‐related high‐mobility group box gene 17 protein (SOX17) is crucial in reconstituting the endothelial cell barrier after injury, normalizing leukocyte transmigration, and mediating hemodynamic adaptability to fluid shear stress.^8^, ^9^ More than a decade ago, SOX17 was found to be expressed in endothelial cells of tumor‐penetrating vessels in murine‐implanted lung carcinoma.^10^ However, the expression profile of SOX17 remains largely unknown in human LUAD.

In the present study, we aimed to unveil the expression of SOX17 in the cancer cells and TME of human LUAD.

## METHODS

### Patient tissue specimens

Archived pathological tissue specimens from 83 cases of LUAD were analyzed in this study. None of the patients received neoadjuvant chemotherapy or radiotherapy or exhibited distant metastasis at diagnosis. This study was performed in accordance with the ethical standards of the Declaration of Helsinki 1975. The use of tissue samples and review of clinical records was approved by the Institutional Review Board of the Gifu University Graduate School of Medicine (approval number: 2024‐053).

### Immunohistochemical staining

All tissue specimens were surgically obtained, fixed in 10% buffered formalin, and embedded in paraffin. The expressions of SOX17 and CD8 were verified by double immunohistochemical staining, as previously reported.^11^ We preliminarily assayed several commercially available specific antibodies against SOX17. Based on the comprehensive study of Shaker et al., on SOX17 expression in normal tissue using rabbit monoclonal antibody against human SOX17 (clone no. EPR20684),^12^ we employed this clone (cat no. AB224637, Abcam, Cambridge, MA, USA) in the present study. Tissue specimens of ovarian high‐grade serous carcinoma were used as positive controls in SOX17 immunohistochemical staining, as they have been reported to be expressed in the same by Shaker et al.^12^

Murine monoclonal antibody against CD8α (clone no. C8/144B; cat no. 70306) was purchased from Cell Signaling Technology (Beverly, MA, USA). The tissues were subjected to immunostaining with antibodies using the MACH2 Double Stain polymer detection kit (Biocare Medical, Concord, CA, USA). Additionally, we performed immunostaining on the tissue specimens using a specific antibody against CD20 (SY12‐01, cat. no. MA5‐32050, Thermo Fisher Scientific, Waltham, MA, USA) following a previously reported protocol.^13^

### Evaluation of immunohistochemical staining

We evaluated SOX17 immunoreactivity in stromal niches and cancer cells. In the former, while SOX17 immunoreactivity was evident in TECs in positive cases, it was not observed in other stromal cells, including fibroblasts, myofibroblasts, and inflammatory cells, in the tissues examined. We thus focused on evaluating SOX17 immunoreactivity in TECs in stromal niches. SOX17‐immunoreactivity was considered positive if TECs exhibited SOX17 immunoreactivity in >10% cells after examining three high‐power fields (400×) per tissue section for each case. SOX17 immunoreactivity was designated as low or high if <10% or >10% of cancer cells were positive.

Regarding CD8 immunoreactivity, specimens were considered “CD8^+^ T‐cell rich” if the specimens harbored the cancer stroma contained more than 50 CD8 immunoreactive cells per high‐power field, while those with fewer were considered “CD8^+^ T‐cell poor.” The number of tertiary lymphoid structures (TLS) was calculated using the immunohistochemical staining method described by Vanhersecke et al. for CD20.^14^ In brief, TLS was considered the presence of a germinal center or the aggregate of more than 50 CD20‐positive B cells.

### Cells and culture

A549 and VMRC‐LCD lung cancer cell lines were obtained from the Japan Health Science Research Resources Bank (Osaka, Japan). The PC‐9 lung cell line was obtained from the Riken Cell Bank (Ibaraki, Japan). Cells were grown in Dulbecco's modified Eagle's medium (Gibco BRL, Life Technologies, Grand Island, NY, USA) with 10% heat‐inactivated fetal bovine serum. The cells were passaged for no more than 6 months after revival.

### Immunofluorescence staining

The detailed procedure described earlier was followed.^15^ In brief, cell clot tissue specimens were incubated with 1 μg/mL of rabbit monoclonal anti‐SOX17 antibody at 25°C for 1 h. After washing with PBS, cells were incubated with goat Alexa Fluor 488‐conjugated anti‐rabbit antibody (1:200) (cat. No. 4412; Cell Signaling Technology, Inc., Dallas, TX, USA) at 25°C for 30 min. After washing, images were acquired using a confocal laser scanning microscope (Leica TCS SP8, Leica Microsystems, Wetzlar, Germany). DAPI (4′,6‐diamidino‐2‐phenylindole) blue staining was used to visualize the nuclei.

### Immunoblot analysis

Immunoblotting was performed following a previously described method with slight modifications, as described in Towbin et al.^16^, ^17^ In brief, proteins were subjected to sodium dodecyl sulfate‐polyacrylamide gel electrophoresis, and separated proteins were electroblotted onto polyvinylidene difluoride membranes (Immobilon‐P Transfer Membrane; Millipore, Bedford, MA, USA). The membranes were blocked with Block Ace (blocking milk; Yukijirushi, Sapporo, Japan) and incubated with 0.5 μg/mL rabbit monoclonal antibody against SOX17 and reprobed by incubation with 0.5 μg/mL murine monoclonal antibody against HDAC1 (Sigma‐Aldrich, St. Louis, MO, USA). Chemiluminescent signals were detected on an Invitrogen iBright 1500 gel imaging system (Thermo Fisher Scientific).

### Statistical analysis

Statistical analysis was performed as previously described.^18^ The relationship between SOX17 and CD8 immunoreactivity was examined using the chi‐squared test. Overall and disease‐free survival curves were generated using the Kaplan–Meier method, and differences in the survival rates were compared using the log‐rank test. Multivariate analysis using the Cox proportional hazards model was used to identify significant prognostic factors. Statistical analyses were performed using EZR version 1.41 (Saitama Medical Center, Jichi Medical University, Saitama, Japan) and a graphical user interface for R (The R Foundation for Statistical Computing, Vienna, Austria).

The association between SOX17 expression and the number of TLS was evaluated by Student's *t*‐test. A *p*‐value of <0.05 was considered significant.

## RESULTS

### Immunoreactivity of SOX17 in LUAD tissue specimens

**FIGURE 1:**
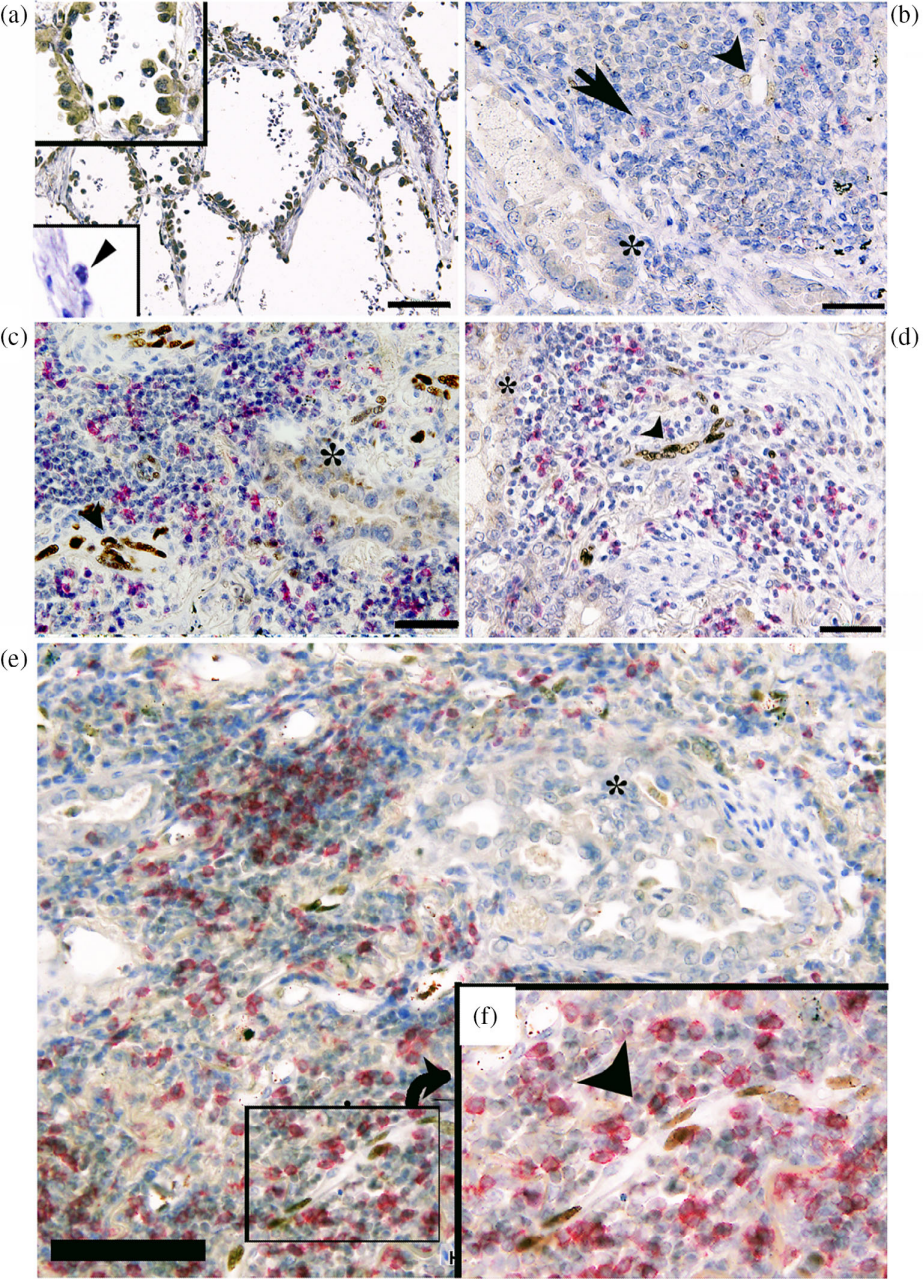
Representative double immunohistochemical staining using a specific antibody against SOX17 (brown) and CD8 (red) in LUAD tissue specimens. (a) SOX17 immunoreactivity (brown) was observed in cancer cells of five noninvasive lung adenocarcinomas harboring morphological similarity with type II alveolar epithelial cells. Note the strong nuclear staining in cancer cells (upper inset). In contrast, noncancerous type II alveolar epithelial cells indicated by the arrowhead did not show significant SOX17 immunoreactivity (lower inset). (b) Little or no SOX17 immunoreactivity was observed in LUAD cells (indicated by an asterisk) and tumor endothelial cells (arrowhead) in cancer stroma, where a few CD8^+^ T cells (arrow) were sparsely found. (c–e) Robust SOX17 immunoreactivity was found in tumor endothelial cells (arrowhead) harboring CD8^+^ T‐cell‐rich cancer stroma. Little SOX17 immunoreactivity was found in LUAD cells (asterisk). (f) Note the intense SOX17 immunoreactivity in tumor endothelial cells around the CD8^+^ T cells (arrowhead). Scale bar indicates 100 μm in a and 50 μm in (b–e). LUAD, lung adenocarcinoma; SOX17, sex‐determining region Y‐related high‐mobility group box 17 protein.

Basic clinicopathological features of the patients with LUAD are described in Table 1. Representative immunohistochemical staining is shown in Figure 1. SOX17 immunoreactivity was minimal in all cancer cells, except for five non‐mucinous adenocarcinomas in situ with morphological features of type II alveolar epithelial cells. In these SOX17‐positive cases, cancer cells ubiquitously exhibited strong SOX17 immunoreactivity (Figure 1a). Notably, SOX17 immunoreactivity was not observed in the lepidic growth area of invasive adenocarcinomas.

**TABLE 1:** Summary of the clinicopathological characteristics of patients based on tumor endothelial SOX‐17 expression.

Features	Total	SOX17‐high	SOX17‐low	*p*	*χ* ^2^
Sex				0.26	1.27
Male	40	7	33		
Female	43	12	31		
Age (years)				0.28	1.18
<70	44	8	36		
≥70	39	11	28		
Histological type				0.58	1.09
In situ	9	1	8		
Invasive	68	16	52		
Invasive mucinous	6	2	4		
Tumor site				0.22	0.63
Right	53	13	40		
Left	30	6	24		
Clinical stage				0.40	5.1
0	9	1	8		
I	54	12	42		
II	5	1	4		
IIIA	11	5	6		
IIIB	3	0	3		
IVA	1	0	1		
Local advance				0.72	2.1
Tis	9	1	8		
T1	48	13	35		
T2	18	4	14		
T3	5	1	4		
T4	3	0	3		
Lymph node metastasis				0.34	2.2
N0	66	13	53		
N1	7	2	5		
N2	10	4	6		
Lymphatic invasion				0.47	0.53
Ly0	58	12	46		
Ly1	25	7	18		
Vascular invasion				0.83	6.0
V0	58	13	45		
V1	24	6	18		
V2	1	0	1		
Stromal CD8^+^				0.00019	13.9
Rich	19	10	9		
Poor	64	8	56		

Abbreviation: SOX17, sex‐determining region Y‐related high‐mobility group box 17 protein.

In contrast, SOX17 immunoreactivity was undetectable in noncancerous bronchial type II alveolar cells, as reported by Shaker et al.^12^ (Figure 1a lower inset). SOX17 immunoreactivity was found in endothelial cells of tumor‐penetrating vessels in 19 of 83 tissue specimens (Figure 1b–f). Eighteen of 83 LUAD specimens appeared to have the CD8^+^ T‐cell‐rich cancer stroma.

As summarized in Tables 1 and 2, SOX17 immunoreactivity was significant in LUAD, harboring a CD8^+^ T‐cell‐rich cancer stroma compared with cases of CD8^+^ T‐cell poor stroma (*p* < 0.01).

**TABLE 2:** Correlation of SOX17 expression in endothelial cells of tumor‐penetrating vessels with CD8^+^ T cells in the cancer stroma.

	CD8^+^ T‐cell‐rich stroma	CD8^+^ T‐cell‐poor stroma
SOX 17 positive	**10**	**9**
SOX 17 negative	**8**	**56**

*Note*: As described in Methods, This relationship between SOX17 and CD8 immunoreactivity was examined using the chi‐squared test. Also as described in Result, SOX17 immunoreactivity was significant in LUAD, harboring a CD8^+^ T‐cell‐rich cancer stroma compared with cases of CD8^+^ T‐cell poor stroma (*p* < 0.01).

Abbreviation: SOX17, sex‐determining region Y‐related high‐mobility group box 17 protein.

Notably, several TECs that demonstrated the morphological features of high endothelial venules (HEVs) exhibited SOX17 immunoreactivity to various degrees (Suppl. Figure 1a). Double immunofluorescent staining using anti‐SOX17 and CD34 (clone QBEnd/10, Dako‐Agilent, Santa Clara, CA, USA) antibodies revealed CD34 expression in SOX17‐positive HEVs (Suppl. Figure 1b,c). HEVs are known to express glycoproteins, including sialyl‐Lewis‐X‐modified CD34, to facilitate activated CD8^+^ T lymphocyte migration.^19^ We thus speculate that SOX17 expression may be related to HEV generation in tumor‐penetrating vessels. Further examination to unravel the pathobiological properties of heterogeneous SOX17 expression in TECs is ongoing.

### SOX17 expression in cultured A549 cells

**FIGURE 2:**
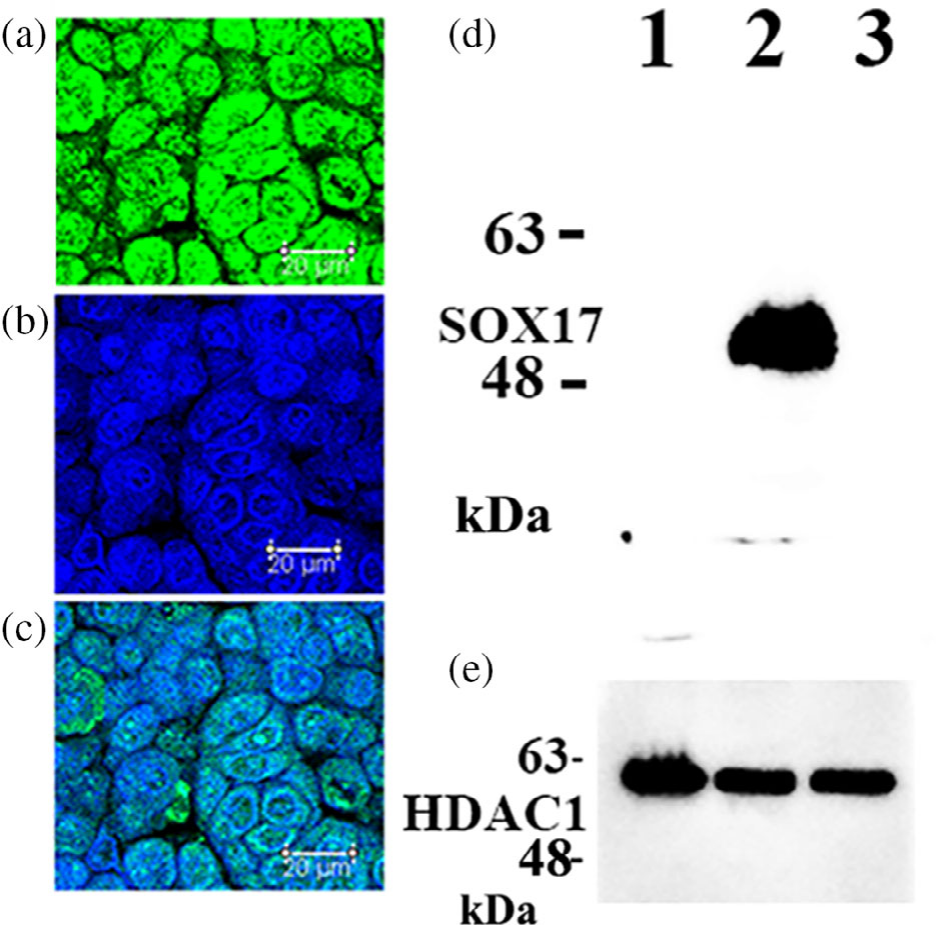
Immunofluorescence staining using antibodies specific to SOX17 (green) (a) and nuclear DAPI (blue) counterstaining (b). Merged cyan nuclear staining was found in the nuclei of A549 lung adenocarcinoma cells (c). Scale bar indicates 20 μm. The immunoblotting procedure revealed a strong SOX17 protein band in A549 cells (d; lane 2). In contrast, the SOX17 protein band was not observed in VMRC‐LCD (d; lane 1) and PC‐9 (d; lane 3) lung cancer cell lines. Protein bands of the internal control, HDAC1, are shown in e. DAPI, 4′,6‐diamidino‐2‐phenylindole; SOX17, sex‐determining region Y‐related high‐mobility group box 17 protein.

As demonstrated in Figure 2, SOX17 was expressed in cultured LUAD A549 cells, which are malignant alveolar type II epithelial cells. In contrast, SOX17 expression was absent in VMRC‐LCD and PC‐9 cells (Figure 2).

### Association between SOX17 immunoreactivity in tumor‐penetrating vessels and TLS

**FIGURE 3:**
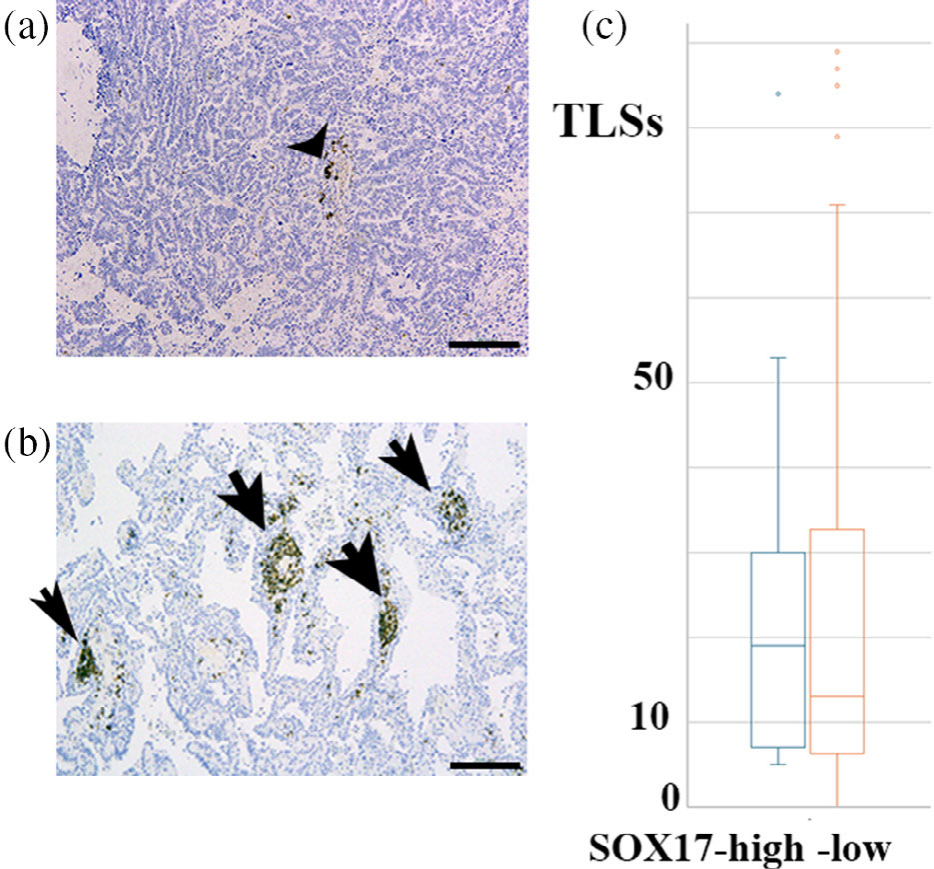
Representative immunohistochemical staining using a specific antibody for CD20 in LUAD tissue specimens. In (a), no lymphoid follicles with germinal centers or clusters of more than 50 CD20‐positive cells was observed; therefore, this case was considered to lack tertiary lymphoid structures (TLS) in this area (arrowheads indicate CD20‐positive cells, but less than 50). In (b), four TLS (indicated by arrow) were observed in this area. Scale bar indicates 200 μm. The total number of TLS within the tumor and within the 5‐mm peritumoral area in each case was counted. The box and whisker plots of TLS in SOX17‐high and low cases. The box represents the 25–75th percentiles, while whiskers show the minimum and maximum values. The horizontal line in the box represents the median number of TLS. There are no statistically significant differences in the number of TLS between SOX‐high (mean 22.6, standard deviation 19.8) and ‐low LUAD (mean 22.8, standard deviation 23.6) (*p* = 0.98). LUAD, lung adenocarcinoma; SOX17, sex‐determining region Y‐related high‐mobility group box 17 protein.

TLS could serve as a niche for CD8^+^ T cells, thereby exerting an antitumor effect.^20^ We subsequently examined whether SOX17 immunoreactivity was related to the number of TLS. As summarized in Figure 3, no significant differences were observed in the number of TLS between SOX17 high and low cases. The influx of CD8^+^ T cells through SOX17‐expressing TECs might be independent of TLS in cancer stromal niches.

### Clinicopathological features of SOX17 immunoreactive tumor‐penetrating vessels

**FIGURE 4:**
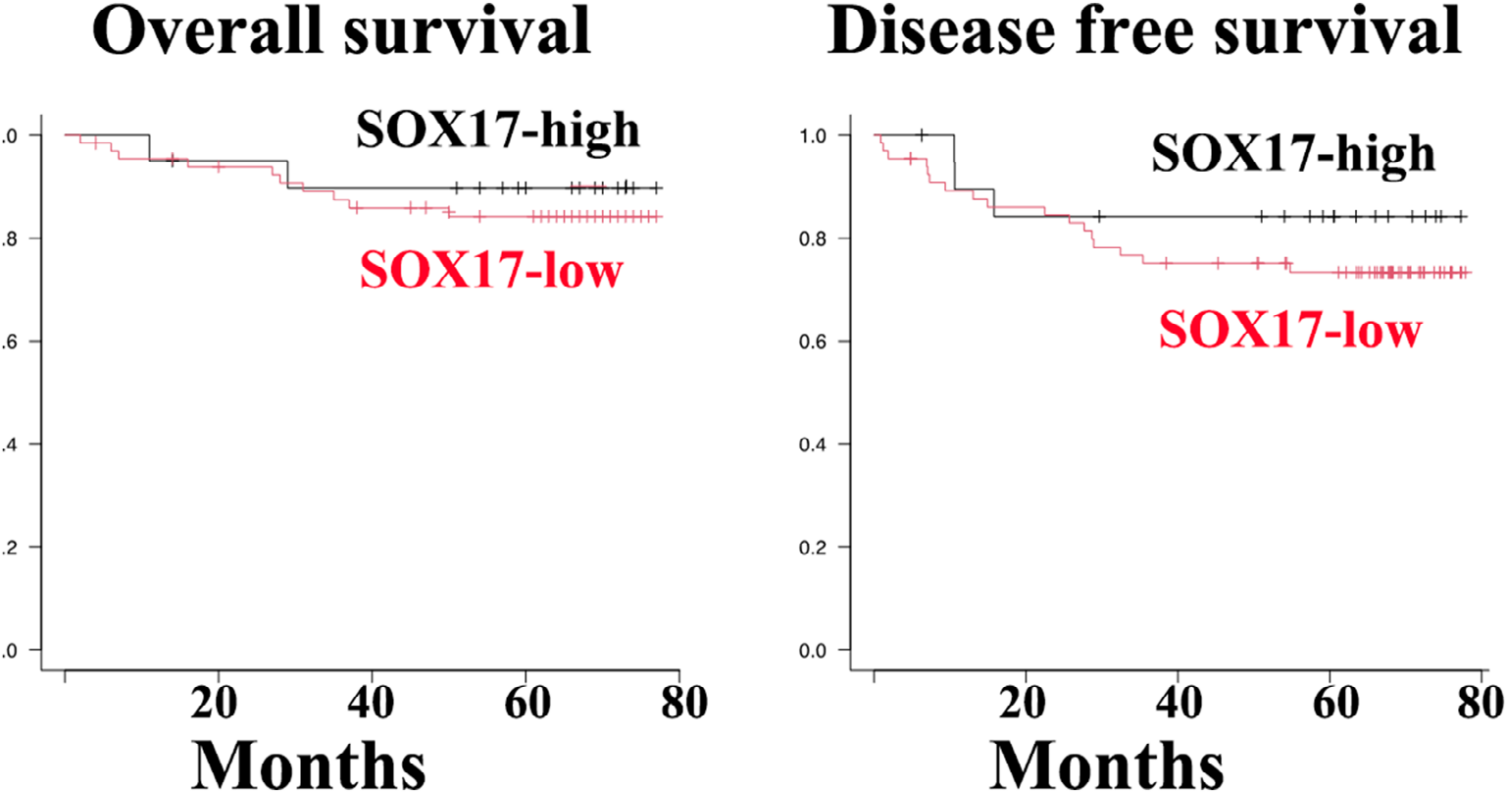
Overall survival and disease‐free survival curves based on SOX17 immunoreactivity in endothelial cells of tumor‐penetrating vessels of LUAD. Both overall and disease‐free survival rates of patients with SOX17‐high immunoreactivity in tumor‐penetrating vessels were more common in patients with LUAD with a favorable outcome than in patients with SOX17‐low LUAD; however, the difference was not statistically significant (overall survival rate, *p* = 0.567; disease‐free survival rate, *p* = 0.364). LUAD, lung adenocarcinoma; SOX17, sex‐determining region Y‐related high‐mobility group box 17 protein.

Kaplan–Meier survival curves indicated that the overall and disease‐free survival rates of patients with tumor endothelial SOX17 immunoreactivity were more favorable than those of SOX17‐negative groups but without statistically significant differences (Figure 4).

## DISCUSSION

In the present study, we examined SOX17 expression in whole tissue specimens by immunohistochemistry, rather than microarray tissue specimens of LUAD. In five of the 83 LUAD tissue specimens, cancer cells exhibited SOX17 immunoreactivity (Figure 1a). Notably, all SOX17 immunoreactivity‐positive LUAD were non‐mucinous adenocarcinoma in situ with alveolar type II cell features. In these cases, cancer cells ubiquitously showed strong SOX17 immunoreactivity, whereas noncancerous alveolar type II cells did not exhibit significant SOX17 immunoreactivity (Figure 1a). We examined SOX17 expression in cultured LUAD cells. As demonstrated in Figure 3, SOX17 was expressed in A549 cells, a widely used cell to model the phenotype of alveolar type II cells.^21^, ^22^ SOX17 is involved in the immune‐evasive process during the early stages of colorectal carcinogenesis.^23^ Accordingly, we speculate that SOX17 may contribute to immune evasion in early non‐mucinous adenocarcinoma in situ; however, extensive experimental proof is needed to verify this hypothesis by examining SOX17 expression in the precancerous lesion, such as atypical bronchioloalveolar cell hyperplasia of the lung.

Regarding SOX17 expression in cancer stroma niches, SOX17 immunoreactivity was found in endothelial cells of tumor‐penetrating vessels in 19 of 83 LUAD cases (Figure 1b–f). SOX17 is well known to play a crucial role in angiogenesis, including that of tumors.^8^, ^9^, ^10^, ^24^, ^25^, ^26^ SOX17 deletion in TECs inhibited tumor angiogenesis in implanted murine Lewis lung carcinoma model.^10^ In human malignant tumors, SOX17 was reported to be predominantly and specifically expressed in TECs in four out of five high‐grade human glioblastoma tissue specimens.^10^ Depleting SOX17 from human high‐grade serous ovarian carcinoma cells altered the expression of factors involved in angiogenesis and functionally disrupted tubule and capillary formation in cell culture and mouse models.^27^ Combined with the present findings of SOX17 immunoreactivity in TECs in 19 of 83 LUAD cases, we reasonably speculate SOX17's role in tumor angiogenesis in various human cancers.

SOX17 promotes the recruitment of inflammatory cells into tumors.^10^ We found significant SOX17 immunoreactivity in TECs in CD8‐positive T‐cell‐rich stroma than in CD8‐positive T‐cell‐poor stroma without association with TLS (Figures 2 and 3). SOX17 expression might facilitate CD8^+^ T‐cell migration to the TME in LUAD. Its expression can be modulated to increase the effect of immunotherapy in patients with advanced LUAD. We plan to perform a clinicopathological study to assess the relationship of SOX17 expression in TECs with the prognosis of the patients receiving immune checkpoint inhibitor therapy, if any.

In conclusion, our three key findings suggest the pleiotropic properties of SOX17 in the carcinogenesis of LUAD. First, SOX17 expression in LUAD cells was limited to non‐mucinous adenocarcinomas in situ. A549 cells showed strong SOX17 expression. Second, SOX17 expression was found in TECs in 19 out of 83 LUAD tissue specimens, showing a significant association with CD8‐positive T‐cell‐rich stroma but not TLS. Third, although statistically insignificant, SOX17 immunoreactivity was related to favorable patient outcomes.

## AUTHOR CONTRIBUTIONS

Conceptualization, Y. H. and T. T.; methodology, C. S.; investigation, H. Y. and Y. H.; formal analysis, Y. H. and T. T.; resources, Y. H. and H. I.; writing – original draft, Y. H.; writing – review and editing, T. T.; visualization, C. S; supervision, T. T. and H. I.; funding acquisition, Y. H. and T. T.

## CONFLICT OF INTEREST STATEMENT

The authors declare no conflicts of interest.

## Supporting information


**Suppl. Fig. 1.** SOX17 immunoreactivity in HEVs. SOX17 immunoreactivity was observed in tumor endothelial cells with HEV features, including a plump, almost cuboidal appearance of varying degrees (a). SOX17 immunoreactivity was nonuniform and rather heterogenous even in adjacent endothelial cells (black and white arrows indicate robust and weak SOX17 immunoreactivity, respectively). The numerous red‐stained CD8^+^ T lymphocytes present around the SOX17‐positive HEVs are notable.
Several tissues were double stained with rabbit monoclonal anti‐SOX17 and murine monoclonal anti‐CD30 antibodies, followed by incubation with goat Alexa Fluor 488‐conjugated anti‐rabbit antibody (1:200) (cat. No. 4412; Cell Signaling Technology, Inc., Dallas, TX) and goat Alex Flour 555‐conjugated anti‐mouse IgG (1:200) (cat. no. A21422; Life Technology, Inc., OR). Alexa Fluor 488‐conjugated anti‐rabbit antibody was adsorbed against mouse serum at Cell Signaling Technology, Inc. Alex Flour 555‐conjugated anti‐mouse IgG was adsorbed against rabbit IgG in our laboratory. The red CD34 immunoreactivity in SOX17‐positive tumor endothelial cells is indicated by the white arrow. SOX17 immunoreactivity is demonstrated as a merge of SOX17‐green and DAPI‐blue. Bar = 20 μm.

## Data Availability

The datasets generated during and/or analyzed during the current study are available from the corresponding author on reasonable request.
