# Supplementary figures and images for: SOX17 expression in tumor‐penetrating vessels in relation to CD8 + T‐cell infiltration in cancer stroma niches
Source: Thorac Cancer. 2024 Oct 9;15(32):2319–26. doi: 10.1111/1759-7714.15464 (PMC11554551; doi:10.1111/1759-7714.15464)

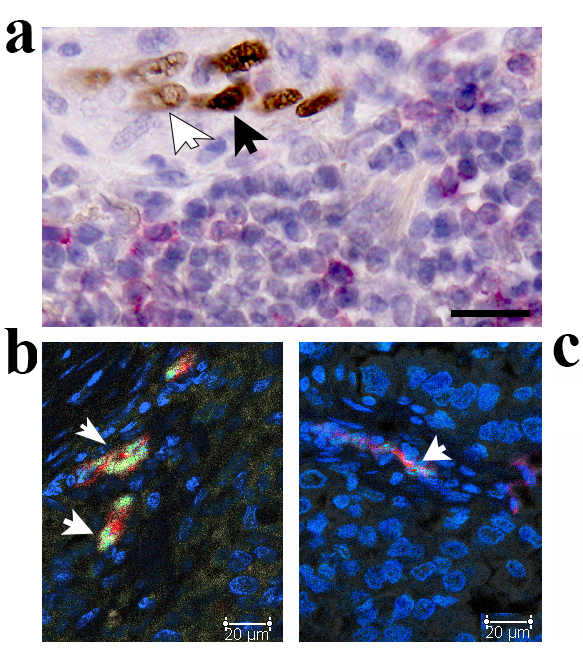

Supplement: Supplementary file 1 — Suppl. Fig. 1. SOX17 immunoreactivity in HEVs. SOX17 immunoreactivity was observed in tumor endothelial cells with HEV features, including a plump, almost cuboidal appearance of varying degrees (a). SOX17 immunoreactivity was nonuniform and rather heterogenous even in adjacent endothelial cells (black and white arrows indicate robust and weak SOX17 immunoreactivity, respectively). The numerous red‐stained CD8+ T lymphocytes present around the SOX17‐positive HEVs are notable. Several tissues were double stained with rabbit monoclonal anti‐SOX17 and murine monoclonal anti‐CD30 antibodies, followed by incubation with goat Alexa Fluor 488‐conjugated anti‐rabbit antibody (1:200) (cat. No. 4412; Cell Signaling Technology, Inc., Dallas, TX) and goat Alex Flour 555‐conjugated anti‐mouse IgG (1:200) (cat. no. A21422; Life Technology, Inc., OR). Alexa Fluor 488‐conjugated anti‐rabbit antibody was adsorbed against mouse serum at Cell Signaling Technology, Inc. Alex Flour 555‐conjugated anti‐mouse IgG was adsorbed against rabbit IgG in our laboratory. The red CD34 immunoreactivity in SOX17‐positive tumor endothelial cells is indicated by the white arrow. SOX17 immunoreactivity is demonstrated as a merge of SOX17‐green and DAPI‐blue. Bar = 20 μm. [file TCA-15-2319-s001.tif]
